# Biofibres from biofuel industrial byproduct—*Pongamia pinnata* seed hull

**DOI:** 10.1186/s40643-017-0144-x

**Published:** 2017-02-21

**Authors:** Puttaswamy Manjula, Govindan Srinikethan, K. Vidya Shetty

**Affiliations:** Department of Chemical Engineering, National Institute of Technology Karnataka, Surathkal, India

**Keywords:** Cellulose microfibres, *Pongamia pinnata* seed hull, Hemicellulose, Lignin, Chlorination

## Abstract

**Background:**

Biodiesel production using *Pongamia pinnata (P. pinnata)* seeds results in large amount of unused seed hull. These seed hulls serve as a potential source for cellulose fibres which can be exploited as reinforcement in composites.

**Methods:**

These seed hulls were processed using chlorination and alkaline extraction process in order to isolate cellulose fibres. Scanning electron microscopy (SEM), dynamic light scattering (DLS), thermogravimetric analysis (TGA), X-ray diffraction (XRD), Fourier transform infrared spectroscopy (FTIR) and nuclear magnetic resonance spectroscopy (NMR) analysis demonstrated the morphological changes in the fibre structure.

**Results:**

Cellulose microfibres of diameter 6–8 µm, hydrodynamic diameter of 58.4 nm and length of 535 nm were isolated. Thermal stability was enhanced by 70 °C and crystallinity index (CI) by 19.8% ensuring isolation of crystalline cellulose fibres.

**Conclusion:**

The sequential chlorination and alkaline treatment stemmed to the isolation of cellulose fibres from *P. pinnata* seed hull. The isolated cellulose fibres possessed enhanced morphological, thermal, and crystalline properties in comparison with *P.* *pinnata* seed hull. These cellulose microfibres may potentially find application as biofillers in biodegradable composites by augmenting their properties.

## Background

Cellulose is nature’s most lavishly available polymer. Highly purified cellulose fibre is been isolated from several plant sources, such as branch barks of mulberry (Li et al. 2009), pineapple leaf fibres (Cherian et al. 2010; Mangal et al. 2003), pea hull fibre (Chen et al. 2009), coconut husk fibres (Rosa et al. 2010), banana rachis (Zuluaga et al. 2009), sugar beet (Dinand et al. 1999; Dufresne et al. 1997), wheat straw (Kaushik and Singh 2011), palm leaf sheath (Maheswari et al. 2012), *Arundo donax* L stem (Fiore et al. 2014), cotton stalk (Hou et al. 2014).

From the past two decades these biofibres are being used as filler material in the preparation of composites and have gained prodigious attention (Hubbe et al. 2008). In view of better utilization of renewable resources, there is a need to explore other renewable greener sources, which can be utilized in developing high strength light weight biocomposites for high-end applications. *Pongamia pinnata* seed hull is chosen for the present work to exploit its potential for cellulose fibres which could be utilized as reinforcement in biocomposites. In India and south East Asia, *Pongamia pinnata* (Karanja) seed is used for biodiesel production (Demirbas 2009). It is also a traditional medicinal plant with all parts having certain medicinal value (Yadav et al. 2004). Biofuel production using *P. pinnata* seeds has resulted in large-scale cultivation of these trees (Shwetha et al. 2014). The biofuel processing fallouts in significant amount of residual *P. pinnata* seed hull, in which cellulose percentage approximates to 40% and is similar as in shelly wood (Nadeem et al. 2009). Thus these underused seed hulls can find potential application as a source for cellulose fibres.

Isolation of cellulose fibres is customarily carried out by mechanical treatments such as homogenisation (Du et al. 2016; Julie et al. 2016), sonication, (Sheltami et al. 2012; Saurabh et al. 2016), steam explosion (Saelee et al. 2014) etc.; chemical treatments such as acid hydrolysis (Abidin et al. 2015), TEMPO oxidation (Du et al. 2016), chlorination and alkaline treatments (Sheltami et al. 2012; Johar et al. 2012; Maheswari et al. 2012) etc.; enzymatic treatments (Saelee et al. 2014) and conjointly with the combination of two or more of the aforementioned processes. Chemical treatments usually act upon the binding material of the fibril structure enabling the fibres to individualize (Johar et al. 2012). Chlorination treatment being a chemical treatment is a well-established treatment which assists isolation of high quality pure cellulose fibres by bleaching and delignifies the cellulose material, while alkali treatment dissolves the wax, pectin and hemicellulose ensuring efficient isolation of cellulose microfibres. These chemical methods are used in combination to isolate cellulose fibres from different sources (Espino et al. 2014; Johar et al. 2012; Sheltami et al. 2012; Mandal and Chakrabarty 2011; Moran et al. 2008) and are also found to be efficient and economical when compared to high energy-consuming mechanical methods (Motaung and Mtibe 2015).

In the present research work, the cellulose fibres were isolated from the *P. pinnata* seed hull using chlorination and alkaline process. The isolated cellulose microfibres were characterized using scanning electron microscopy (SEM), dynamic light scattering (DLS), thermogravimetric analysis (TGA), X-ray diffraction (XRD), Fourier transform infrared spectroscopy (FTIR) and nuclear magnetic resonance spectroscopy (NMR) analysis for their morphological, thermal and crystalline properties.

## Methods

### Materials

*Pongamia pinnata* seed hulls were collected from “SEEDS” Research Centre, University of Agricultural Sciences, Bengaluru, India. All the chemicals used were of analytical grade.

### Fibre processing

*Pongamia pinnata* seed hulls were separated from stones and other plant materials by hand picking. The dust and mud particles sticking to the seed hulls were removed by washing them extensively in tap water and finally with distilled water. Later dried under sunlight for two days and stored in sealed polythene bags for further use. Cleaned seed hulls were ground, screened (0.25 mm sieves) and oven dried at 105 °C for 8 h.

### Isolation of cellulose microfibres

Cellulose microfibres were isolated from *P. pinnata* seed hull by chlorination and alkaline extraction process (Maheswari et al. 2012). Cleaned seed hull fibres were dewaxed using toluene–ethanol mixture (2:1) for 6 h. Excess of solvent from the fibres was removed by suction and later kept for drying in hot air oven. Fibres were bleached with 7% NaClO_2_ taken in fibre to liquor ratio of 1:50 (pH vicinity 4–4.2 was maintained using acetic acid and sodium acetate buffer) for 2 h at 100 °C and was washed successively using 2% sodium bisulphate, distilled water and ethanol. Further the extraction of holocellulose from fibres was carried out by treating with 17.5% NaOH solution at 20 °C for 45 min and subsequently washed with 10% acetic acid. Later the fibres were treated with 0.8% acetic acid and 0.7% nitric acid in the ratio 15:1 at 120 °C for 15 min. The mixture was cooled, filtered and washed sequentially with 95% ethanol and distilled water. The resulting cellulose fibres were oven dried at 105 °C until consistent weight was achieved.

### Characterization

#### Scanning electron microscopy (SEM)

The morphological structure of gold-sputtered *P. pinnata* seed hull fibres and isolated cellulose fibres were observed under SEM (JSM-6380LA, JEOL, EVISA). The micrographs were recorded at acceleration voltage of 5–8 kV.

#### Dynamic light scattering (DLS)

The fibre dimension of aqueous dispersed isolated cellulose fibre (distilled water) was measured by the dynamic light scattering instrument (DLS, nanoparticle analyser, HORIBA Scientific, nano partica SZ-100, Japan).

#### Fourier transform infrared spectroscopy (FTIR)

*Pongamia pinnata* seed hull fibres and isolated cellulose fibres mixed with KBr were pressed to form transparent thin pellets. FTIR spectra of the fibres were recorded in the extent of 400–4000/cm with 4/cm resolution using FTIR instrument (Jasco 4200, Jasco analytical instruments, USA).

#### X-ray analysis (XRD)

XRD measurements for *P. pinnata* seed hull fibres and isolated cellulose fibres were obtained by X-ray diffractometer (X’Pert^3^ Powder, PANalytical, The Netherlands) using Cu Kα radiation (1.5406 Å) with Ni filtered at 40 kV, 15 mA. Scattered radiations were recorded in the range of 2*θ* = 10° − 30° at a scan rate of 4°/min. The Segal method [Eq. (1)] was used to calculate crystallinity index (CI) considering the intensities of $\left( {2 0 0} \right)$ peak (*I*_200_, 2*θ* = 22.6°) and the intensity minimum between the $(2 0 0)$ and $(1 1 0)$ peaks (*I*_am_, 2*θ* = 18^0^), where *I*_200_ represents the intensities of crystalline and amorphous material and *I*_am_ for the amorphous material.

1 $${\text{CI}} \% = \left( {1 - \frac{{I_{am} }}{{I_{200} }}} \right) \times 100$$

#### Thermogravimetric analysis (TGA)

Thermograms for *P. pinnata* seed hull fibres and isolated cellulose fibres were determined using a thermogravimetric analyser (TGA Q50, TA instruments, USA) at a 10 °C/min heating rate in nitrogen atmosphere.

#### ^13^C NMR (CP-MAS) spectroscopy

Spectra of *P. pinnata* seed hull fibres and isolated cellulose fibres were run on solid-state NMR spectrometer (Bruker DSX 300 MHz). 75.46 MHz operating frequency was fixed for ^13^C nuclei. Fibres were spun at 7.5 kHz spinning rate with filled 5 mm rotor at room temperature.

## Results and discussion

Delignification of seed hull using acidified sodium chlorite was compassed, as an initial step in the isolation of cellulose. The alkaline treatment aids in the oxidation of lignin and hemicellulose, solubilizes the residual lignin and hemicellulose resulting in the isolation of cellulose fibres. These cellulose fibres were characterized for their morphological features, thermal stability and also to ensure removal of matrix components such as lignin and hemicellulose.

### SEM analysis

**Fig. 1:**
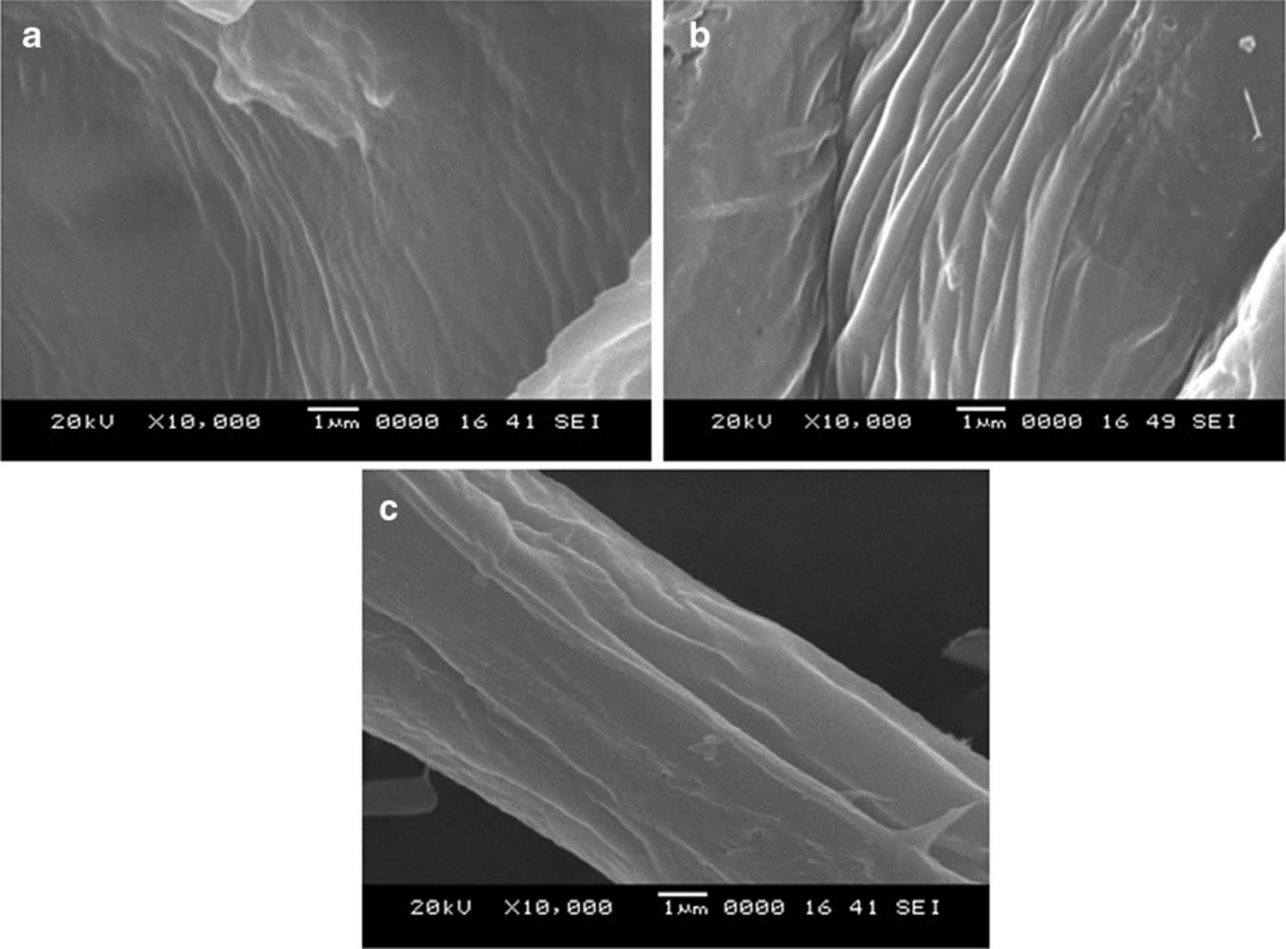
Scanning electron microscope images **a** dewaxed *Pongamia pinnata* seed hull, **b** sodium chlorite-treated fibres and **c** isolated cellulose fibres

The scanning electron microscope images of *P. pinnata* seed hull fibre after different stages of chemical treatment are as presented in Fig. 1a–c. Dewaxed seed hull fibre presented in Fig. 1a show irregular appearance due to cellulose fibre embedded between waxes and cementing materials such as lignin and hemicellulose (Reddy and Yang 2005; Haafiza et al. 2013). The fibres after sodium chlorite bleaching show cellulose fibres emerging out of the matrix as shown in Fig. 1b. This could be accounted to oxidation and solubilisation of matrix components viz. lignin and hemicellulose. The cementing components—lignin and hemicellulose isolated from the fibres are dissolved by mild alkali treatment (Elanthikkal et al. 2010). As a result, the SEM image of the isolated cellulose fibres as presented in Fig. 1c illustrates individualized single strand of cellulose fibres of diameter 6–8 µm, which in turn is a bundle of cellulose microfibres (Chen et al. 2011) having diameter of 270–370 nm. The cellulose fibres isolated in this work were of smaller diameter compared to that of the other cellulose fibres obtained from different sources such as soybean straw (Reddy and Yang 2009) yielding fibres of diameter 15.6 µm and coconut palm sheath (Maheswari et al. 2012) yielding fibres of 10–15 µm diameter.

### DLS analysis

**Fig. 2:**
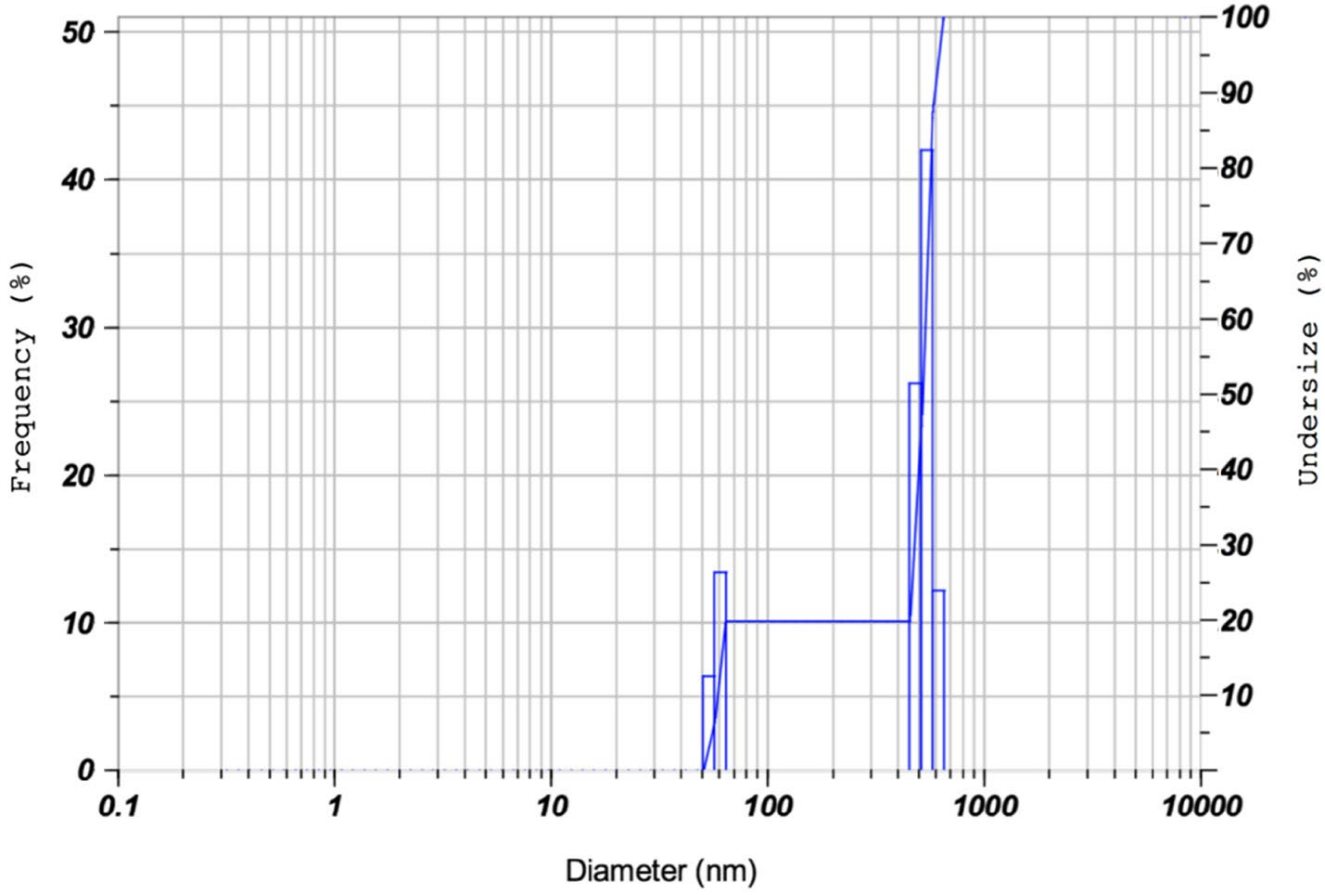
DLS analysis spectra of isolated cellulose fibres

The aqueous dispersion of cellulose fibres was analysed by the dynamic light scattering technique in order to find their size distribution. DLS analysis results are summarized in Table 1. The histogram presented in Fig. 2 shows the presence of two distributions, indicating the presence of two dimensions (Kavitha et al. 2013; Srinivas et al. 2012) which is owing to the fibrous structure of cellulose representing both length and diameter. de Carvalho Mendes et al. (2015) also reported such two peaks in DLS histogram of the aqueous dispersion of cellulose fibrous structure. Dimensions determined by DLS epitomise hydrodynamic size (sphere size) having same diffusional coefficient as the fibres being measured (Horiba knowledgebase 2017). The mean hydrodynamic size of isolated cellulose fibres for shorter dimension (diameter) was observed to be 58.4 nm, whereas the longer dimension (length) of the fibres was observed to be 536.3 nm with the standard deviation of 3.3 and 44.1 nm, respectively. The diameter of the fibre obtained by SEM analysis is lesser than that obtained by DLS technique. The sizes of the fibre obtained by SEM and DLS are not comparable, as the diameter obtained by SEM presents the dry fibre size, whereas that obtained by DLS signifies the hydrodynamic diameter in aqueous dispersion. The difference in size estimated by the two methods is generally higher for the nonspherical particles.

**Table 1:** Particle size distribution values of isolated cellulose fibre

Peak No.	S.P. area ratio^a^	Mean (nm)	SD^b^ (nm)	Mode (nm)
1	0.20	58.4	3.3	58.9
2	0.80	536.3	44.1	535.0
Total	1.00	441.9	194.3	535.00

^a^Specific particle surface area ratio

^b^Standard deviation

### Fourier transform infrared spectroscopy (FTIR)

**Fig. 3:**
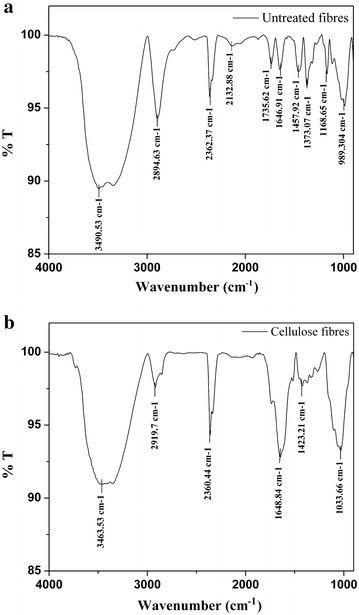
FTIR spectra of **a** *Pongamia pinnata* seed hull fibres, **b** isolated cellulose fibres

FTIR spectroscopy monitors the functional groups present in the fibres. Figure 3a and b present the spectra obtained for *P. pinnata* seed hull fibres and isolated cellulose fibres. The band around 3600–3000/cm assigned to stretching vibrations of O–H and C–H is observed in both *P. pinnata* seed hull fibres and isolated cellulose fibre, indicating the presence of cellulose-related functional groups (Qiao et al. 2016; Shin et al. 2012; Kalita et al. 2015; Sun et al. 2004a, b, c; Kaushik and Singh 2011). Peaks at 2894.63 and 2919.7/cm is generally assigned to C–H stretching vibration in lignin polysaccharide (cellulose and hemicellulose) (Shin et al. 2012; Sun et al. 2004a, b, c, d; Kaushik and Singh 2011; Zhong et al. 2013). Peak at 1735.62/cm is assigned to C=O stretching vibration of carbonyl, acetyl and uronic ester group of the ferulic and p-coumaric acids of lignin and/or xylan component of hemicellulose. The disappearance of these peaks in cellulose fibre spectra, confirms the removal of lignin and hemicellulose (Kalita et al. 2015; Kaushik and Singh 2011; Sun et al. 2004a, c, d; Elanthikkal et al. 2010; Rosa et al. 2012; Oun and Rhim 2016). Peaks at 1646.91 and 1648.84/cm are attributed to O–H bending of absorbed water and are observed in both the spectra; the presence of water could be related to the hydrophilic nature of cellulose component even though the samples analysed were dry (Qiao et al. 2016; Sun et al. 2004b, c; Kaushik and Singh 2011; Zhong et al. 2013; Rosa et al. 2012; Oun and Rhim 2016; Haafiza et al. 2013). Peaks at 1457.92 and 1423.21/cm are usually attributed to aromatic C=C stretch of lignin and the reduction of peak at 1423.21/cm in cellulose fibre spectra indicates the fractional delignification after the treatments (Sun et al. 2004a, b, c, d; Kaushik and Singh 2011; Elanthikkal et al. 2010; Haafiza et al. 2013). Peaks around 1373.07 and 1168.65/cm observed in *P. pinnata* seed hull fibres are assigned to C–H asymmetric deformation and C–O antisymmetric bridge stretching, respectively (Kalita et al. 2015; Sun et al. 2004a, b, c, d; Kaushik and Singh 2011; Zhong et al. 2013; Rosa et al. 2012). Finally the increase in peak 1033.66/cm, observed in isolated cellulose fibre spectra attributed to –C–O–C– pyranose ring skeletal vibration which indicates an increase in cellulose content (Sun et al. 2004a, b; Elanthikkal et al. 2010).

#### ^13^C NMR (CP-MAS) spectroscopy

**Fig. 4:**
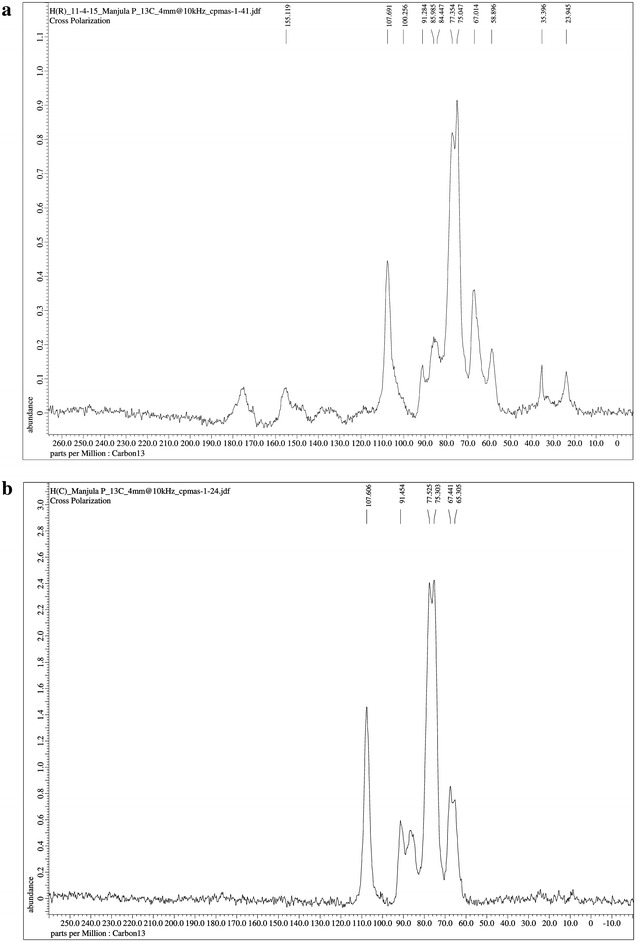
The ^13^C NMR spectra of **a** untreated *Pongamia pinnata* seed hull fibres, **b** isolated cellulose fibres

The ^13^C NMR spectra of untreated *P. pinnata* seed hull fibres and isolated cellulose are as shown in Fig. 4a, b. *P. pinnata* seed hull fibres spectrum in Fig. 4a, illustrates the presence of corresponding signals for the cellulose, hemicellulose and lignin, whereas in the case of isolated cellulose fibre spectrum as shown in Fig. 4b peaks of only cellulose carbon atoms were illustrated. Peaks between 107 and 60 ppm corresponding to six carbon atoms assigned to cellulose molecules are observed in both the spectra. The cellulose carbon atom peak at 107.6 is associated with C1 (Halonen et al. 2013), peaks at 77–67 ppm are assigned to C2, C3 and C5 carbon atoms (Sun et al. 2004a, d), peaks at 91.454 − 84.447 are of C4 (Bhattacharya et al. 2008) and finally 65.305 − 58 is associated with C6 carbon atom (Sun et al. 2004b, c, d). Similar observations were reported by Halonen et al. 2013, where the peaks around 109 − 101 ppm were associated with C1 atom, 80 − 68 ppm to C2, C3 and C5, 91 − 80 ppm to C4 and 68 − 58 ppm to C6 (Bhattacharya et al. 2008). In case of cellulose spectrum, the absence of peaks at 20–33 and 110–140 ppm associated with methylenes in lignin and 58.896 ppm of –OCH_3_ groups in lignin and hemicellulose, ensures the removal of hemicellulose and lignin, the matrix components (Sun et al. 2004d; Bhattacharya et al. 2008).

Thus the removal of hemicellulose and lignin from the *P. pinnata* seed hull fibres are supported by both NMR and FTIR spectral data.

#### Thermogravimetric analysis (TGA)

**Fig. 5:**
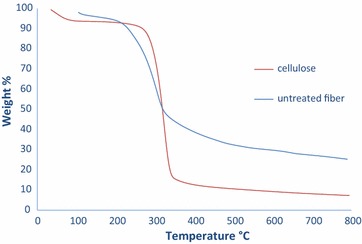
Thermogram of untreated *Pongamia pinnata* seed hull fibres and isolated cellulose fibres

The thermograms of untreated *P. pinnata* seed hull fibres and isolated cellulose fibres as shown in Fig. 5 have onset degradation temperature of 200 and 270 °C, respectively. The major degradation peak at around 250–350 °C observed for isolated cellulose fibre is mainly due to pyrolysis of cellulose and thermal depolymerisation of hemicellulose (Abraham et al. 2011; Li et al. 2015; Chen et al. 2011; Luduena et al. 2011), showing 75% degradation of cellulose. The increase in the decomposition temperature of the isolated cellulose fibres is related to the crystallinity of cellulose due to the removal of lignin and amorphous hemicelluloses (Abe and Yano 2009). Residual presence in both *P. pinnata* seed hull fibres and isolated cellulose fibres at 800 °C was observed to be 25 and 7%, respectively, which indicates reduction in the presence of carbonaceous materials in the nitrogen atmosphere which is associated with the removal of hemicellulose (Li et al. 2015). Thus the high thermal properties perceived in case of isolated cellulose microfibres may broaden the fields of application of cellulose fibres at temperatures above 200 °C especially for biocomposite processing.

### X-ray diffraction (XRD)

**Fig. 6:**
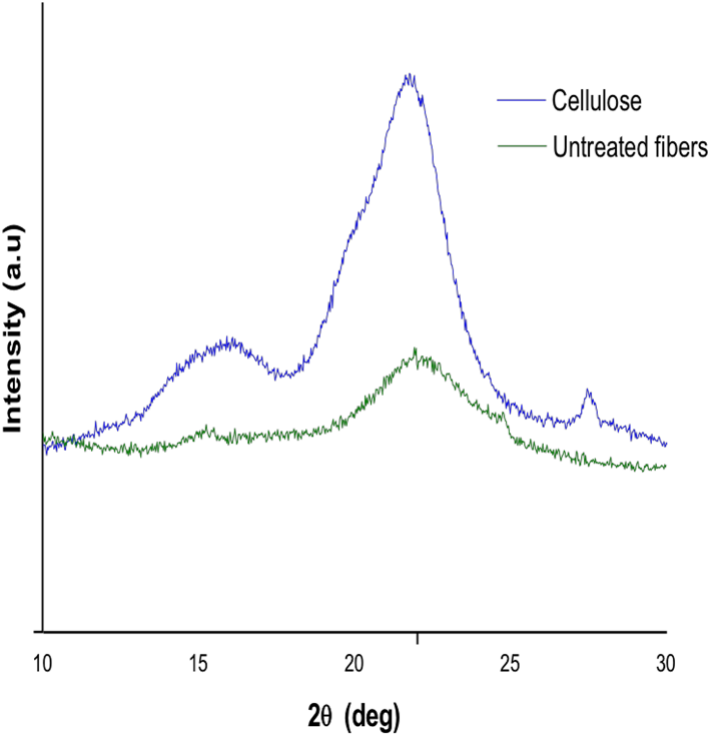
X-ray diffraction patterns of untreated *Pongamia pinnata* seed hull and isolated cellulose fibres

X-ray diffractograms of *P. pinnata* seed hull fibres and isolated cellulose fibres are presented in Fig. 6. Two peaks are observed at 2*θ* = 16° and 22.6° for both the samples which is the characteristic of crystal polymorphs of cellulose I and cellulose II, respectively (Bondeson et al. 2006; Novo et al. 2015). The peak at 2*θ* = 16° corresponds to the $(1 1 0)$ and 2*θ* = 22.6° corresponds to the $(2 0 0)$. The crystallinity index (CI) obtained using Eq. (1) for *P. pinnata* seed hull fibres and isolated cellulose fibres were 27.2, and 47%, respectively. The crystallinity of the isolated cellulose microfibres was increased by 72.79%. This could be due to the presence of large amount of crystalline cellulose and removal of amorphous hemicellulose and lignin (Rosa et al. 2010) from isolated cellulose fibres by chlorination and alkaline treatment.

Thus, from the above results it can be observed that cellulose microfibres isolated from *P. pinnata* seed hull exhibited enhanced morphological, thermal and crystalline properties after chlorination and alkaline treatment. Size and increase in crystallinity of the cellulose fibres obtained from different sources and isolation methods are summarized in Table 2. The size of the fibres obtained in the present work is comparable with that obtained from other sources by different isolation methods. However, percentage increase in crystallinity for the fibres isolated from *P. pinnata* seed hull after chlorination and alkaline treatment is higher than that for the fibres isolated from other sources by chemical treatment methods obtained by other researchers. As observed in Table 2, increase in crystallinity is lower in most of the cases in spite of additional mechanical treatments. Julie et al. (2016) have obtained around 97% increase in crystallinity of the fibres isolated from Arecanut husk fibres. However, they have adopted homogenization, a mechanical process after chemical treatment. Isolation of cellulose microfibres by chlorination and alkaline treatment is economical compared to others, as enormous amount of energy is consumed in the mechanical treatments. The chlorination and alkaline treatment on *P. pinnata* seed hull resulted in the isolation of crystalline cellulose fibres of 6–8 μm diameter. It is observed that the cellulose fibres isolated from *P. pinnata* seed hull show higher percentage increase in crystallinity when compared to cellulose fibres obtained from other resources by chemical treatments. Higher crystallinity of cellulose fibres accounts to higher tensile strength of the fibres (Alemdar and Sain 2008), which in turn is expected to enhance the mechanical properties of the cellulose fibre-reinforced composites.

**Table 2:** Comparison of fibre size and crystallinity index (CI) of cellulose fibre isolated from different sources and isolation treatments

S. no	Source	Size (diameter) of cellulose fibres as observed under SEM	Crystallinity index (CI) isolated cellulose fibre (%)	Increase in crystallinity (%)	Treatment	Reference
1	Resak’s hardwood waste	7–12 (μm)	68.1	37.33	Alkaline and acid hydrolysis	(Abidin et al. 2015)
2	Corn husk	5–8 (nm)	63.3	46.5	Alkaline, TEMPO oxidation, and homogenization	(Du et al. 2016)
3	Arecanut husk fibre	3–5 (nm)	73	97	Alkaline, acid hydrolysis, Bleaching (Chlorination), homogenization	(Julie et al. 2016)
4	Moso bamboo culms	0.5–1 (μm)	65.32	28.87	Microwave liquefaction, bleaching (Chlorination), Alkaline, homogenization and ultrasonication	(Xie et al. 2016)
5	*Gigantochloa scortechinii* bamboo culms	5.29–10.94 (nm)	65.32	36.33	Acid hydrolysis, homogenization, sonication	(Saurabh et al. 2016)
6	Sugarcane bagasse	<20 (μm)	–	–	Steam explosion, enzymatic treatment	(Saelee et al. 2014)
7	Mengkuang leaves	5–80 (μm)	69.5	26.13	Alkaline Bleaching (Chlorination), sonication	(Sheltami et al. 2012)
8	Rice husk	7 (μm)	59	26.06	Alkali, bleaching	(Johar et al. 2012)
9	Coconut palm leaf sheath	10–15 (μm)	47.7	12.7	Chlorination and alkaline	(Maheswari et al. 2012)
10	*Pongamia pinnata* seed hull	6–8 (μm)	47	72.79	Chlorination and alkaline	Present work

## Conclusion

Cellulose fibres were isolated from *P. pinnata* seed hull by sequential chlorination and alkaline process and the resultant microfibres were characterized by SEM, DLS, FTIR, NMR, TGA and XRD analyses. Cellulose microfibres were in diameter ranging from 6 to 8 μm and mean hydrodynamic diameter of 58.4 nm. NMR and FTIR analyses confirmed the removal of hemicellulose and lignin. Crystallinity of the fibres was increased by 72.79% after the treatment with CI of 47% for the isolated cellulose fibres. Thermal behaviour of the fibres had improved as evidenced by an increase of degradation temperature by 70 °C. Most potential observation to be considered was the degradation temperature of the isolated cellulose fibres being higher than 200 °C, which could broaden its application potential in the fields of biocomposite processing. A notable increase in crystallinity and the dimension similar to the cellulose fibres isolated from other resources by various chemical treatments was a significant feature of the resource and the isolation method adopted in the present study. Thus the present work substantiates the success of the sequential chlorination and alkaline extraction process solely contributing to obtain smaller diameter and crystalline cellulose microfibres from *P. pinnata* seed hull. These biofibres have potential application as filler, embroiling in the process of biodegradable composites to enhance their properties.
